# A phase II study of the insulin-like growth factor type I receptor inhibitor IMC-A12 in patients with metastatic uveal melanoma

**DOI:** 10.1097/CMR.0000000000000694

**Published:** 2020-09-21

**Authors:** Jane Mattei, Alexej Ballhausen, Roland Bassett, Michael Shephard, Chandrani Chattopadhyay, Courtney Hudgens, Michael Tetzlaff, Scott Woodman, Takami Sato, Sapna P. Patel

**Affiliations:** a Department of Melanoma Medical Oncology; b Department of Biostatistics; c Department of Translational and Molecular Pathology; d Department of Genomic Medicine, The University of Texas MD Anderson Cancer Center, Houston, Texas, USA; e Department of Applied Tumor Biology, Institute of Pathology, University Hospital Heidelberg, Heidelberg, Germany; f Department of Medical Oncology, Thomas Jefferson University, Philadelphia, Pennsylvania, USA

**Keywords:** insulin-like growth factor type I receptor, phase II, uveal melanoma

## Abstract

Uveal melanoma is a rare and aggressive malignancy and up to half of all patients will develop metastatic disease despite the effective treatment of the primary tumor. Insulin-like growth factors I/II play a fundamental role in the cell migration, proliferation, and apoptosis. IMC-A12, a mAb specifically targets insulin-like growth factor type I receptor, has shown promise in preclinical studies. We performed a multicenter phase II study for patients with metastatic uveal melanoma administered IMC-A12 10 mg/kg IV every two weeks until disease progression or unacceptable toxicity. The primary endpoint was objective response (proportion of patients with complete or partial response), and secondary endpoints were disease control rate, progression-free survival, and overall survival. A total of 18 patients enrolled in this study (10 males and eight females) with a median age. Ten patients (55%) had stable disease, seven patients (38%) had progression as best overall response. No partial response or complete response was observed; however, the disease control rate, defined as complete response + partial response + stable disease ≥3 months, was 50%. Median progression-free survival was 3.1 months, and median overall survival was 13.8 months. Adverse events of any grade occurred in 13 patients (72.2%). Treatment-related grade 3 adverse events were rare, and there were no grade 4 or 5 related adverse events. IMC-A12 was very well tolerated, however, showed limited clinical activity in uveal melanoma as a single agent. Due to its low toxicity profile it could be studied in combination with other pathway-specific agents.

## Background

Uveal melanoma is the most common primary intraocular malignant tumor in adults and the second most common type of primary malignant melanoma in the body. Although metastatic disease is rare at the time of diagnosis, approximately 50% of patients will eventually develop distant disease, despite definitive treatment for their primary uveal melanoma with brachytherapy, enucleation, or proton beam radiation. Most commonly, metastatic disease develops through vascular spread to the liver, which accounts for 95% of metastatic cases. Patients who develop distant disease have a poor prognosis and despite therapy, their median survival is six to seven months with a dismal one-year survival of approximately 10–15%. Locoregional treatment options for metastatic disease include local surgical resection, radiofrequency ablation, or chemoembolization [1,2]. Systemic chemotherapy has failed to show any significant efficacy against metastatic uveal melanoma. Retrospective reviews from MD Anderson Cancer Center, Eastern Cooperative Oncology Group and Southwest Oncology Group indicated that patients with uveal melanoma (especially those with liver metastasis) rarely responded to drugs commonly used for the treatment of metastatic cutaneous/mucosal melanoma [2,3].

So far, no specific targeted therapies have proven activity in uveal melanoma, but preclinical studies suggest potential benefit of inhibitors of c-mesenchymal to epithelial transition (c-Met), sarcoma (SRC), rat sarcoma (Ras), epidermal growth factor receptor (EGFR), and vascular endothelial growth factor (VEGF) receptor tyrosine kinases, phosphatidylinositol-3-kinase-AKT pathways, and receptor tyrosine kinases and insulin-like growth factor type I receptor (IGF-1R) [4,5]. IGF-1R is a member of the insulin receptor family and is activated through binding of two high-affinity ligands [6]. While insulin receptor substrate-1 (IGF-1) is believed to mediate the mutagenic effects of IGF-1R, insulin receptor substrate-2 (IGF-2) is believed to play a role in metabolic and proliferative signals triggered by the insulin receptors. Primary pathways involved in IGF transduction are the phosphatidylinositol 3-kinase/Akt and mitogen-activated protein kinase pathways [7,8].

IMC-A12 or Cixutuxumab (Imclone, Inc.) is a recombinant human IgG1 mAb which specifically targets the human IGF-1R and antagonizes IGF-1 and IGF-2 ligand binding and signaling, while it does not bind or recognize the human insulin receptor [9,10]. In addition, binding of IMC-A12 to IGF-1R leads to internalization and degradation of the receptor. IMC-A12 inhibits the proliferation and growth of a variety of human tumor cell lines, both *in vitro* and *in vivo* [2].

In two phase I studies, IMC-A12 was evaluated for safety and antitumor effects either weekly (CP13-0501; total of 24 patients enrolled) or every other week (CP13-0502; total of 16 patients enrolled) at doses ranging from 3 mg/kg through 27 mg/kg. Common adverse event included hyperglycemia, while severe adverse events occurred infrequently. No maximum tolerated dose was identified. Dosing with ≥6 mg/kg weekly and ≥10 mg/kg every-2-week, predetermined target serum minimum concentrations (60 μg/mL) were achieved. Overall, stable disease was the best response in 25% of all patients.

IGF-1R expression has been shown to significantly correlate with worse prognosis in uveal melanoma patients [4,11,12]. We hypothesized that this increased mortality rate may be due to Akt activation through the IGF-1 pathway. Thus, targeting IGF-1R with IMC-A12 may have the potential to control metastasis in uveal melanoma patients. We found IMC-A12 to be effective in target inhibition, in blocking IGF-1R dependent signaling and cell migration in multiple UM cells that we tested.

## Patients and methods

### Patient eligibility

Patients with history of uveal melanoma and histologically confirmed metastatic disease. Patients were required to be at least 17 years old, have an Eastern Cooperative Oncology Group performance status of 0–2, life expectancy of at least three months, at least one measurable lesion >10 mm according Response Evaluation Criteria in Solid Tumors (RECIST v1.1). Laboratory values within the prespecified range for absolute neutrophil count (≥1500/mm^3^), platelets (≥100 000/mm^3^), hemoglobin (≥90 g/L), serum creatinine (≤1.5 X institutional ULN), serum total bilirubin (≤1.5 X institutional ULN), aspartate and alanine aminotransferases (≤3 × ULN or ≤5 × ULN for patients with liver metastases), and fasting serum glucose (<120 mg/dL). Patients had a washout period of at least six weeks between the last dose of the most recent prior immunotherapy, cytokine, biologic vaccine therapy, tumor embolization, and four weeks from prior radiotherapy or chemotherapy. We excluded patients with symptomatic brain metastasis requiring steroids, leptomeningeal disease, ascites, pleural/pericardial effusions, carcinomatous lymphangitis, Gilbert syndrome, HIV-positive patients with an absolute CD4 count <300 K/uL, patients with other active neoplasia, pregnant women, patients with serious active infections, and patients with a history of treatment with other agents targeting the IGF pathway. Patients with a history of allergic reactions attributed to compounds of similar chemical or biologic composition to IMC-A12 were also excluded.

The study protocol was conducted at MD Anderson and approved by the MD Anderson Cancer Center Institutional Review Board. Furthermore, the study was done in accordance with the Declaration of Helsinki and International Conference of Harmonization Good Clinical Practice. Informed consent was obtained from all subjects before enrollment.

### Study design and treatment

In this open-label Phase II trial enrolled patients were assigned to receive 10 mg/kg IV infusion over 1 hour every two weeks with treatment cycle defined as four weeks. Dose reduction was allowed according to protocol. Patients continued treatment until disease progression, unmanageable toxicity, termination of study, or death. We did disease assessment scans at baseline and then every eight weeks until discontinuation of study treatment. Response and progression were evaluated using the new international criteria proposed by the RECIST criteria (version 1.1). We used the National Cancer Institute Common Toxicity Criteria (CTCAE version 4.0) to grade adverse events. The primary endpoint of the study was investigator-assessed objective response rate (proportion of patients with complete or partial response). Secondary endpoints were to determine the disease control rate (the proportion of subjects with a confirmed complete or partial response of any duration or stable disease ≥3 months in duration), progression-free survival (PFS), and overall survival (OS).

### Statistical analysis

A Simon minimax two-stage design was used to assess the primary endpoint of response rate using RECIST 1.0. Based on 90% power and an alpha level of 0.1 and assuming a clinically uninteresting response rate of 5% (null hypothesis H0: *P* ≤ 5%) and a target response rate of at least 20% (alternative hypothesis Ha: *P* ≥ 20%), a total of 32 evaluable subjects (defined as those subjects who received at least one dose of protocol therapy and had at least one post-treatment assessment of target and nontarget lesions) was planned. In Stage 1, 18 evaluable subjects were planned. If at least one response is observed in Stage 1, enrollment in Stage 2 will be initiated. If no response is observed in Stage 1 after observing the last enrolled subject for a minimum of four cycles, enrollment will be stopped. In Stage 2, 14 additional evaluable subjects will be enrolled. If at least four responses are observed among the total number of evaluable subjects enrolled in Stages 1 and 2 combined, the null hypothesis will be rejected. If three or fewer responses are observed among the total number of evaluable subjects enrolled in Stages 1 and 2 combined, the null hypothesis will not be rejected.

In addition, baseline IGF-1R expression via immunohistochemistry was evaluated for correlation to response after the first 6–7 patients treated. This analysis was planned so as to inform conduct of the remainder of the trial and provide for a possible enrichment strategy.

The intent-to-treat population was defined as all subjects registered in the study. The safety population was defined as all subjects who received any amount of protocol therapy in the study. PFS and OS were computed using the methods of Kaplan–Meier.

### Western blot

Cells were lysed in a buffer containing 50 mmol/l Tris (pH 7.9), 150 mmol/l NaCl, 1% NP40, 1 mmol/l EDTA, 10% glycerol, 1 mmol/l sodium vanadate, and protease inhibitor cocktail (Roche Pharmaceuticals, Nutley, New Jersey). Proteins were separated by SDS-PAGE with 4–20% gradient gels (Bio-Rad Laboratories, Hercules, California), transferred to a Hybond-ECL nitrocellulose membrane (GE Healthcare Biosciences, Piscataway, New Jersey), and blocked in 5% dry milk in PBS. The membrane was then incubated with primary and secondary antibodies, and target proteins were detected with ECL detection reagent (GE Healthcare Biosciences).

### Cell migration assay

Assays for uveal melanoma cell migration were performed in Boyden chambers using uncoated filters (BD Biocoat control 5inserts, BD Biocoat, San Jose, California). A 2.5 × 10 cells/well were plated in serum-free medium, with or without a 6-hour treatment of 50 ug/mL IMC-A12, and the migration assay performed [13]. Stained cells were photographed with a Nikon Eclipse TE2000-U microscope at ×20 magnification using NIS Elements advanced research software.

### Immunohistochemistry technique and evaluation

Histopathological review and immunohistochemical stain for IGF-1R [IGF-1Rα Antibody (N-20): sc-712, Santa Cruz Biotechnology, Dallas] using a 1:1000 dilution. Paraffin sections of 4 μm were deparaffinized, rehydrated, and rinsed. After rinsing, the endogenous peroxidase activity was blocked by treatment with 0.5% hydrogen peroxide for 30 minutes. The sections were then rinsed and incubated with blocking serum (1% BSA) for 20 minutes. The IGF-1Rα antibody was applied and incubated overnight at +8° Celsius. A biotinylated anti-rabbit antibody immunoglobulin G was applied as a secondary antibody and incubated for 30 minutes and then rinsed and the avidin-biotin complex incubated for another 30 minutes. The peroxide reaction was developed using 3,3′-diaminobenzidine tetrahydrochloride (0.6 mg/mL with 0.03% hydrogen peroxide) for 6 minutes. Counterstaining was performed by Mayer’s hematoxylin. Tris-buffered saline (pH 7.6) was used for rinsing between the different steps. Normal placenta tissue was used for positive controls [4,11,14].

## Results

### Effect of IMC-A12 on UM cells

**Fig. 1:**
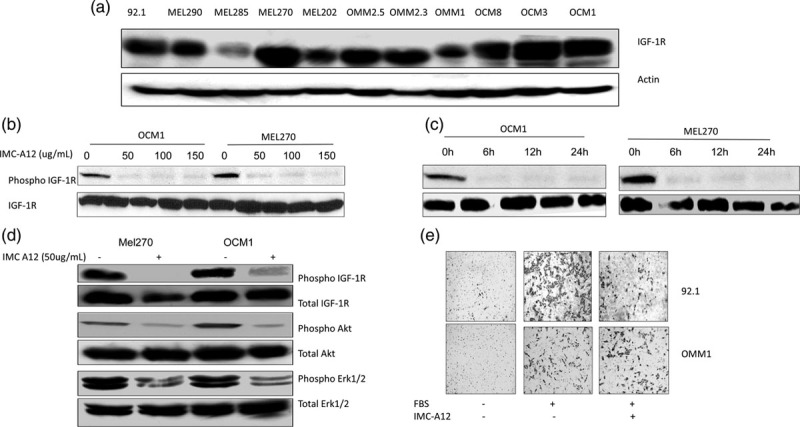
Insulin-like growth factor type I receptor (IGF-1R) expression and effect of IMC-A12 in UM cells. (a) IGF-1R protein levels in a panel of UM cells. (b) IMC-A12 inhibits IGF-1R activation in UM cells: IMC-A12 dose response in UM cells showing inhibition of IGF-1R phosphorylation on treatment of OCM1 and MEL270 with increasing concentration of the agent. (c) Time course of IMC-A12 mediated IGF-1R inhibition: 50 ug/mL IMC-A12 can completely inhibit IGF-1R phosphorylation post 6 h treatment in vitro. (d) IMC-A12 inhibits IGF-1R dependent signaling in UM cells: A treatment with 50 ug/mL IMC-A12 inhibited Akt and Erk1/2 activation in UM cells post 6 h treatment. (e) IMC-A12 inhibits UM cell migration in an in vitro cell migration assay: 50 ug/mL IMC-A12 inhibited cell migration in 92.1 and OMM1 cells on overnight treatment.

In an attempt to characterize the effect of IMC-A12 *in vitro*, we tested the effect of this IGF-1R blocking antibody on inhibition of target activation, downstream signaling, and cell migration of UM cells. First, we determined the expression levels of the target IGF-1R in a panel of UM cell lines with different genetic background[15,16] and found all of them to express the IGF-1R (Fig. 1a). When UM cell lines from this panel were treated with IMC-A12, complete inhibition of IGF-1R phosphorylation/activation was observed at 50 ug/mL IMC-A12 concentration and within 6-hour treatment period (Fig. 1b and c). On testing the effects of IMC-A12 on downstream effectors of IGF-1 signaling, we observed efficient inhibition of Akt and Erk1/2 activation in UM cells (Fig. 1d). Interestingly, testing this antibody in an *in vitro* cell migration assay with UM cells and 10% FBS as chemo-attractant showed differential effects of IMC-A12 in blocking cell migration based on the cell line tested (Fig. 1e). A possible explanation of this selectivity may be due to difference in migration potential of these cell lines used, with cell line 92.1 being highly migratory as observed in upper-middle panel of Fig. 1e as compared to considerably lower migration potential of OMM1(lower middle panel of Fig. 1e).

### Patient characteristics

Between 8 May 2011 and 4 January 2013, 18 patients were enrolled at two separate institutions. The clinical characteristics of these patients are described in (Table 1). The median age was 64 years (range 26–85), and 56% of the study population was male. One patient had type II diabetes mellitus, and one patient had prediabetes before starting the clinical study. The majority of patients had received 0–1 prior therapies for metastatic uveal melanoma.

**Table 1:** Patients’ characteristics

Characteristic	No. of patients (%), n = 18
Median age (range), y	64 (range 26–85)
Gender	
Male	10 (56)
Female	8 (44)
ECOG performance status	
0	10 (56)
1	3 (17)
2	1 (5)
Unknown	4 (22)
Past medical history of prediabetes or diabetes	
Prediabetes	1 (5)
Diabetes	1 (5)
Sites of metastasis	
Liver only	6 (33)
Liver and other sites	9 (50)
Lung	7 (39)
Bone	5 (28)
Pleura	2 (11)
Lymph nodes	2 (11)
Adrenal glands	2 (11)
Peritoneal	2 (11)
Subcutis	2 (11)
Deep soft tissue	2 (11)
Pancreas	1 (5)
Portal vein	1 (5)
Kidney	1 (5)
Bladder	1 (5)
Other sites only	3 (17)
Lung (bilateral)	2 (11)
Spleen	1 (5)
Prior therapy for metastatic disease	
0	7 (39)
1	8 (44)
2	1 (5)
3+	2 (11)

ECOG, Eastern Cooperative Oncology Group.

### Toxicity

Adverse events of any grade occurred in 13 of 18 patients (72.2%). The most common adverse events considered related to treatment were constitutional symptoms such as fatigue (50%) and weight loss (39%) followed by laboratory changes of hyperglycemia (28%) and increased aspartate aminotransferase (28%). Treatment-related grade 3 adverse events were rare and occurred in one patient (5%) each including anorexia, diarrhea, hypertension, dehydration and increased lipase, cholesterol, and triglycerides. There were no grade 4 or 5 related adverse events. A list of treatment-related adverse events occurring in more than one is presented in Table 2.

**Table 2:** Treatment-related adverse events occurring in more than one patient

Adverse events	Grade 1–2 (%)	Grade 3 (%)
Anemia	2 (11.1)	0
Anorexia	2 (11.1)	1 (5.5)
Telangiectasia	2 (11.1)	0
Skin and subcutaneous disorders	2 (11.1)	0
Headache	2 (11.1)	0
Paresthesia	2 (11.1)	0
Hyperkalemia	2 (11.1)	0
Hypocalcemia	2 (11.1)	0
Hyperuricemia	2 (11.1)	0
ALT increased	2 (11.1)	0
Thrombocytopenia	3 (16.6)	0
Pruritus	3 (16.6)	0
Amylase increased	3 (16.6)	0
High cholesterol	3 (16.6)	1 (5.5)
Rash	4 (22.2)	0
Dry skin	4 (22.2)	0
Nausea	4 (22.2)	0
Diarrhea	4 (22.2)	1 (5.5)
Lipase increased	4 (22.2)	1 (5.5)
Hypertriglyceridemia	4 (22.2)	1 (5.5)
Hyperglycemia	5 (27.7)	0
AST increased	5 (27.7)	0
Weight loss	7 (38.8)	0
Fatigue	9 (50.0)	0

ALT, alanine aminotransferase; AST, aspartate aminotransferase.

### Efficacy

**Fig. 2:**
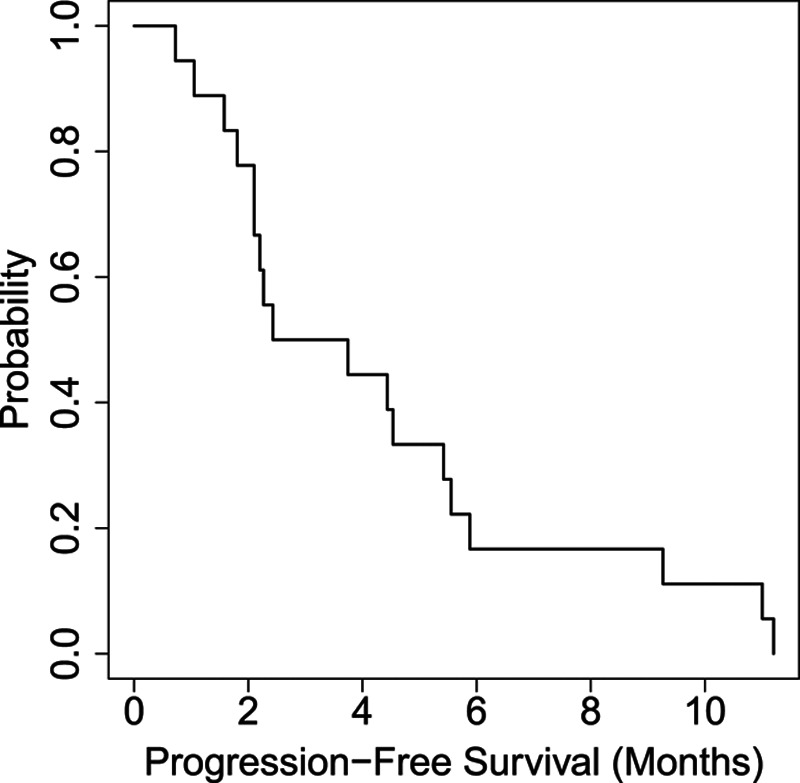
Represents a Kaplan–Meier plot of progression-free survival (PFS). The median PFS was 3.1 months.

**Fig. 3:**
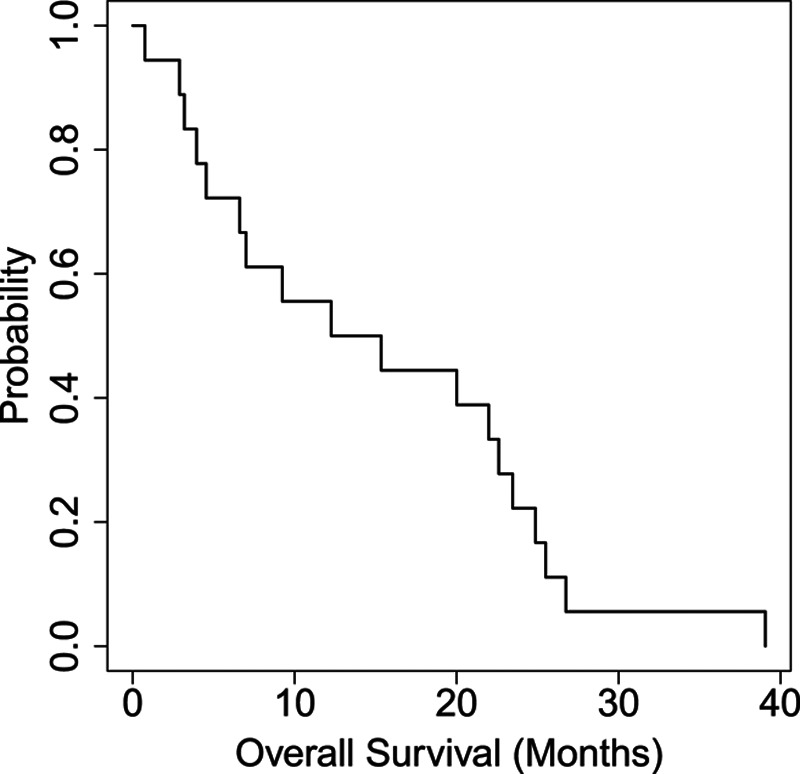
Represents a Kaplan–Meier plot of overall survival (OS). The median OS was 13.8 months.

The median follow-up period was 20 weeks. Of 18 patients treated with IMC-A12, 10 patients (55%) had stable disease as best response, seven patients (38%) had progression of disease, and one patient (6%) was not evaluable for response due to death prior to follow-up scans. No partial response or complete response was observed, however, the disease control rate, defined as complete response + partial response + stable disease ≥3 months, was 50%. All patients were evaluable for PFS and OS analyses. At the time of data analysis, all patients had died. Median PFS was 3.1 months (Fig. 2), and median OS was 13.8 months (Fig. 3).

### Immunohistochemistry for IGF-1R

Baseline metastatic tumor samples were received for 17 patients. Adequate tumor tissue for IGF-1R expression was available in 15 of the 17 cases. Cases were reviewed by a pathologist and scored according to the following percentage of tumor positive for IGF-1R: 1– <33% = 1; 33–<67% = 2; and 67–100% = 3. There was no baseline IGF-1R expression in seven cases (47%); four cases (27%) had a baseline score of 1, while two cases (13%) had a score of 2, and another two (13%) cases had a baseline IGF-1R score of 3.

## Discussion

The IGF pathway is one of many targetable pathways activated in metastatic uveal melanoma. Our study had no clinical responses to single-agent IMC-A12 and a disease control rate of 50%, suggesting half the patients experienced stable disease for at least three months, which was prespecified secondary endpoint per study protocol. However, the median PFS of 3.1 months fails to translate this therapy into a meaningful outcome. One reason for lack of observed clinical activity of IMC-A12 may be related to low or absent IGF-1R expression in the study population. Nearly 75% of the baseline tissues analyzed had absent or 0–<33% tumor expression of IGF-1R. The lack of a highly expressed target is directly related to the lack of efficacy. Additionally, because of the complex nature of signaling networks in uveal melanoma, it is unlikely that a single targeted agent will be able to provide enough growth inhibition to withstand compensatory signaling cascades. Notably, however, IMC-A12 was minimally toxic, with no grade 4 treatment-related adverse events, and low frequency of grade 3 events occurring in one patient each. The patient diagnosed with type II diabetes mellitus was taking glimepiride before starting the protocol. It was not necessary to add any new medication for his diabetes. Compensatory pathways are known to be activated in uveal melanoma, including HGF-c-Met, SRC, Ras, EGFR, VEGF, and phosphatidylinositol-3-kinase-AKT signaling. Moving forward, IGF pathway inhibition owing to its low toxicity profile could be studied in combination with other pathway-specific agents using baseline IGF-1R expression as an entry criterion, and on-treatment biopsies to determine the amount of target saturation or receptor occupancy taking place. To date, there are no single-agent therapies with meaningful efficacy in metastatic uveal melanoma. Well-designed combination trials with correlative studies are needed to fill this knowledge gap.

## Acknowledgements

The authors would like to thank the faculty and staff and patients who participated in the study execution. The study was supported by the Cancer Therapy Evaluation Program (CTEP) under NIH award number N01CM62202 and the NIH/NCI Cancer Center Support Grant under award number P30CA016672.

## Conflicts of interest

There are no conflicts of interest.
